# 4K 3-dimensional video microscope system (orbeye) for transsphenoidal pituitary surgery

**DOI:** 10.1007/s00701-021-04762-x

**Published:** 2021-02-22

**Authors:** Roman Rotermund, Jan Regelsberger, Katharina Osterhage, Jens Aberle, Jörg Flitsch

**Affiliations:** 1 Department of Neurosurgery, University Medical Center Hamburg-Eppendorf, Martinistr. 52, 20246 Hamburg, Germany; 2 Department of Endocrinology, University Medical Center Hamburg-Eppendorf, Martinistr. 52, 20246 Hamburg, Germany

**Keywords:** Pituitary surgery, Transsphenoidal, Digital three-dimensional 4K exoscope, 4K 3D video microscopy, Microsurgery, Orbeye

## Abstract

**Background:**

In previous reports on experiences with an exoscope, this new technology was not found to be applicable for transsphenoidal pituitary surgery. As a specialized center for pituitary surgery, we were using a 4K 3D video microscope (Orbeye, Olympus) to evaluate the system for its use in transsphenoidal pituitary surgery in comparison to conventional microscopy.

**Method:**

We report on 296 cases performed with the Orbeye at a single institution. An observational study was conducted with standardized subjective evaluation by the surgeons after each procedure. An objective measurement was added to compare the exoscopic and microscopic methods, involving surgery time and the initial postoperative remission rate in matched cohorts.

**Results:**

The patients presented with a wide range of pathologies. No serious events or minor complications occurred based on the usage of the 4K 3D exoscope. There was no need for switching back to the microscope in any of the cases. Compared to our microsurgically operated collective, there was no significant difference regarding duration of surgery, complications, or extent of resection. The surgeons rated the Orbeye beneficial in regard to instrument size, positioning, surgeon’s ergonomics, learning curve, image resolution, and high magnification.

**Conclusions:**

The Orbeye exoscope presents with optical and digital zoom options as well as a 4K image resolution and 3D visualization resulting in better depth perception and flexibility in comparison to the microscope. Split screen mode offers the complementary benefit of the endoscope which may increase the possibilities of lateral view but has to be evaluated in comparison to endoscopic transsphenoidal procedures in the next step.

## Introduction

Since its introduction in the 1960s, the surgical microscope has become the gold standard in microneurosurgery enabling the surgeon to operate on even small lesions in deep anatomical locations. A further step was the introduction of endoscopy, either as a standalone or complementary tool. Herewith, transsphenoidal pituitary surgery developed two main surgical approaches: either using the microscopy or endoscopy with technical related benefits and limitations, which are still vigorously debated in the literature [2, 5, 9, 12, 17].

Advances within the digital era now add exoscopes of different designs and with various features to the neurosurgical armamentarium. Comparable to the endoscopic procedures, the surgical field is illuminated and monitored by a 4K digital camera, coupled with LED light. The surgical procedure is controlled and directed via a monitor illustrating the so-called video microscopy. In respect to transsphenoidal approaches, the exoscope combines features of both endoscopy and microscopy: it offers the digital camera monitor system of the endoscopic technique in combination with the extracorporeal positioning and maneuverability similar or even much better compared to the microscope.

For over 10 years [10, 11], different types of exoscopes have been introduced, including pencil-like inspection tools, added by robotic systems, an incorporated navigation, and now 3D and high-resolution camera systems. The latter provides an improved image resolution, color reproduction, illumination, focal length, depth of field, and stereopsis. This has been confirmed to be comparable to the standard of microscopic surgery [8]. Clinical evaluations have shown that a three-dimensional image, 4K resolution, a natural color reproduction, and easy maneuverability are mandatory for fine movements, orientation in the operative field, and identification of anatomical structures, tissue boundaries, lesions, and hemorrhage, and are therefore essential for the operative workflow and clinical outcome [4, 13, 16, 18].

The 4K 3D Exoscope (Orbeye, Olympus) has proven to be suitable for a broad range of neurosurgical procedures such as craniotomies including cerebral bypass surgery [15], cavernous malformations, brain tumor resection, and intracerebral hematoma evacuation [7, 19], as well as for the entire spectrum of spinal microsurgery [6].

It has also been used for transcranial pituitary surgery [20]. However, for a long time, it was not considered to be feasible for transsphenoidal approaches [14]. We now used the 4K 3D exoscope for all transsphenoidal pituitary surgeries after a short trial period and report our experiences with this new system.

## Material and methods

### Specification of the 4K 3D exoscope

The 4K 3D Surgical Camera System (OME-V200; “Orbeye”) consists of a dual-optics camera system mounted on a freely maneuverable, counterbalanced arm, which is attached to the processor-containing base. The light source (OME-L200) consists of an LED panel with the light being transmitted via fiber optics to the camera head. For maneuvering the camera, the surgeon has two options: the exoscope is mainly being controlled manually by pushing a release button on the side of the camera with the middle finger of the leading hand to have a free range of motion. For the fine adjustment of the visual field during surgery, especially when both hands are needed to operate, an additional joystick at the front of the foot pedal can be used, similar to the method generally used in microscopic surgery.

The image is projected to a 31-inch medical grade 4K 3D monitor (SOMED) with a 4160 × 2160 pixel resolution (Full 4K). The optical system has a 1:6 motorized optical zoom, which was fully used. Sometimes the acquired image was ×2 digitally zoomed, resulting in a total zoom of 1:12. The exoscope-specific setting and positioning was arranged in a manner so that the maximum magnified view of ×26 could be achieved. This magnification results from the combination of the 1:12 zoom of the camera head plus the magnification of the size of the monitor at the minimum distance of the camera head to the focal plane and monitor to surgeon, respectively. The optical zoom does not affect the image quality, whereas the digital zoom slightly reduced the quality of the image due to increased size of the pixels but still maintained the detail and color reproduction of the 4K image. For a 3D image, the surgeons wear polarized 3D glasses. A second 4K 3D monitor was positioned behind the surgeon and set to a 2D output for assistants and nursing personnel in the operating room.

### Setting and positioning of patient

**Fig. 1:**
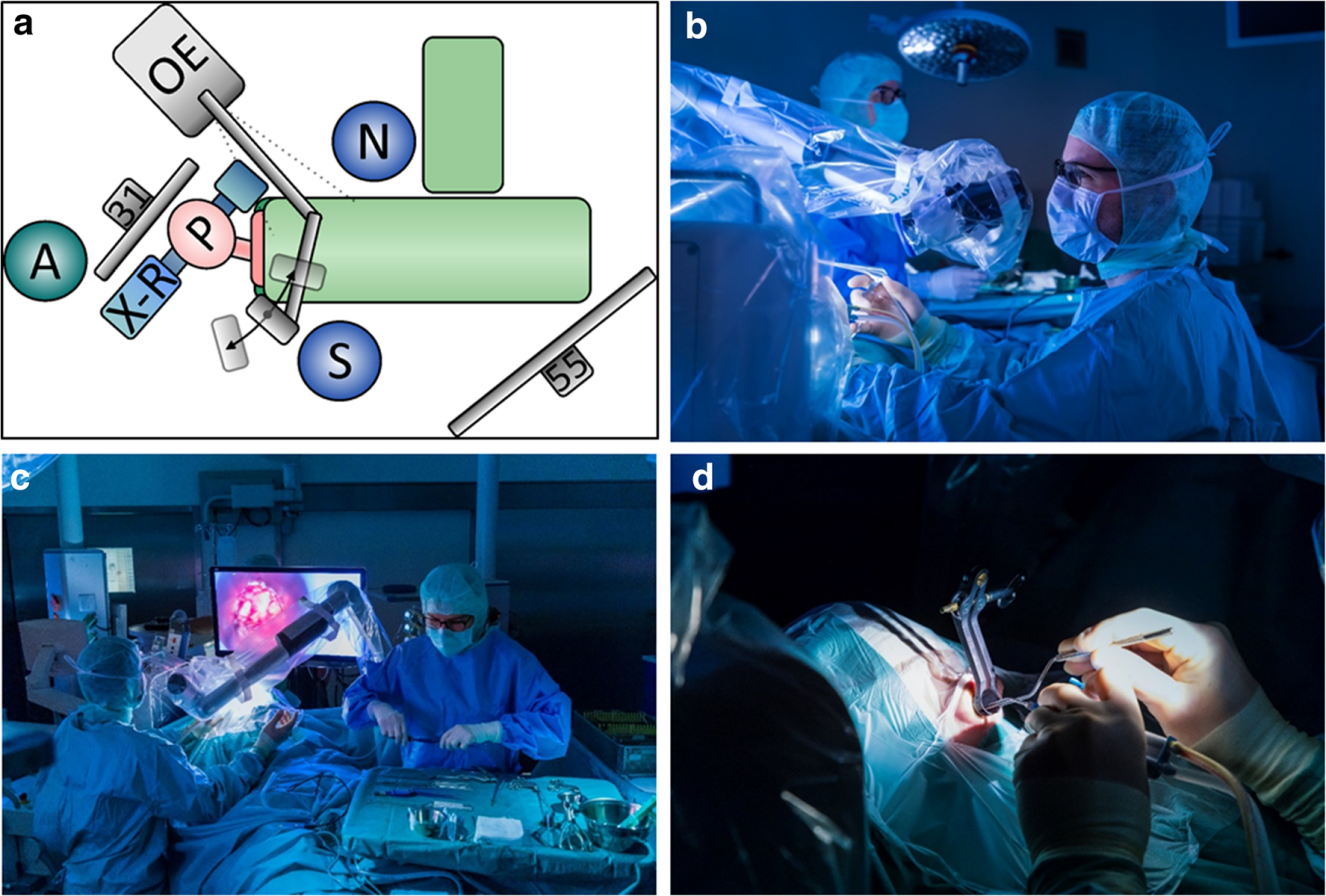
Setting in the operating room for transsphenoidal pituitary surgery with the Orbeye 4K 3D video microscope. **a** Schematic drawing of the positioning of the patient and equipment. The patient (P) is lying in supine position with the head slightly bent upwards and to the right of the surgeon (S). The position of the O. R. nurse (N), Orbeye (OE) with its two monitors, C-arm of the intraoperative x-ray (X-R), and the anesthesiologist (A). **b** Photograph from a lateral view. **c** Photographs from a posterior view. **d** Photograph with a close-up on the operative field

The surgical setup is shown in Fig. 1. The patient is positioned in a semi-sitting supine position. The patient’s head is turned to the right towards the surgeon. The Orbeye is positioned opposite of the surgeon behind the patient’s head with the camera arm reaching over towards the surgeon and the camera head pointing upwards in line with the nasal septum. The camera’s distance to the focal plane of the surgical view was 220 to 300 mm. The 31-inch monitor projecting the 4K 3D image is mounted to a boom-arm reaching down from the ceiling and is positioned above the patient’s head directly in front of the surgeons view at a distance of around 0.5 m. The O.R. nurse is positioned at the patient’s left side in line with the surgeon. A second 55-inch monitor projecting a 2D image is positioned opposite to the first monitor on a mobile monitor stand. The image of the second monitor was turned 180° to ensure the correct orientation of the image for the O.R. nurse. The anesthesiologist is located behind the patient’s head and the first 4K 3D-monitor. An x-ray C-arm is statically positioned around the patient’s head. The lights are dimmed in the operation room during the whole procedure.

### Patients and procedures

Two hundred and ninety-six transsphenoidal operations were performed between 06 February 2019 and 01 April 2020 non-consecutively at a single university medical center institution using the Orbeye system. Median patient’s age was 50 years (6–86 years). The gender distribution was 153 female vs. 143 male. There were no exclusion criteria. The patient cohort shows a wide spectrum of pathologies (Table 1). Seventy-nine of the 296 operated patients (27%) had undergone previous treatment for their disease: 59 × 1–4 transsphenoidal surgeries (of those, six patients additionally have undergone radiation, pharmaceutical therapy, or transcranial surgery before), 17 had undergone only pharmaceutical pretreatments for prolactinomas/acromegaly, and three had undergone bilateral adrenalectomies for Cushing’s disease. The median tumor/cyst size was 1.7 cm × 1.5 cm × 1.5 cm (179 < 1 cm, 117 > 1 cm). The invasiveness of the 239 adenomas was determined with the Knosp’s grading system. All surgeries were conducted by the transsphenoidal approach, analogous to that procedure so far used in our microscopic transsphenoidal pituitary surgeries via monoportal transseptal route (initial mucosal incision at lamina perpendicularis). After opening the anterior wall of the sphenoid sinus, the sella region can be directly addressed.

**Table 1:** Pathologies operated from February 2019 to April 2020

Pathology		*N*
Adenomas, *n* = 239	Non-functioning pituitary adenomas	99
	Acromegaly	67
	M. Cushing	48
	Prolactinomas	21
	TSHoma	3
	PANCH (pituitary adenoma with neuronal choristoma)	1
Craniopharyngioma		12
Rathke’s cleft cyst		12
Colloid cyst		2
Arachnoid cyst		2
Meningioma WHO °I		7
Chordoma		4
Germinoma (biopsies)		3
Pilocytic astrocytoma		3
Adenoidcystic carcinoma		2
NCC metastasis		1
Prostate cancer metastasis		1
Hamartoma with ACTH expression		1
Hypophysitis due to Rathke’s cleft cyst		1
Pituicytoma WHO °I		1
Spindle cell oncocytoma		1
Angioma		1
Ependymoma		1
Histiocytic tumor		1
No histological specimen		2

The surgeries were performed by two senior surgeons (J. F. and R. R.) working as a team or separately. Both surgeons are specialized pituitary surgeons; the distribution was random. In difficult cases, four-eye reception control was performed. Here, the surgeons operated alternately with one surgeon handling the exoscope and the other surgeon observing. A change in the instrument positioning after changing the operating surgeon was not necessary in any case. Our surgeons used the Orbeye exclusively in a sitting position. A standing position is also possible as for example used in spine surgery. The surgeons at our clinic use the foot pedal mainly for focus and zoom as well as adjustment of the light intensity. Other options include switching to NBI and 5-ALA vision.

Overall, the functionality of the Orbeye System was evaluated based on the achievement of the goals set prior to surgery (tumor resection/reduction/nerve decompression/biopsy). In case of adenomas, the results of the surgeries are measured semi-quantitatively with the amount of tumor resected (gross total resection, partial resection) and in the further comparison of hormonally active microadenomas without prior surgical or medical treatment the postoperative biochemical remission was assessed. The personal impression of the surgeon regarding feasibility and utility of the 3D 4K exoscope was collected postoperatively (no categories or fixed questions).

## Results

Two hundred ninety-six surgeries were exclusively performed with the 4K 3D exoscope. The mean duration of the procedure was 1:29 h (0:34–04:43). A switch to the conventional microscope was not necessary in any of the cases. The preoperatively established surgical goal was achieved in all but one of the cases when the approach to a purely suprasellar tumor in a child was terminated due to massive vessel formations at the tumor surface; the procedure was followed by transcranial surgery confirming pilocytic astrocytoma. One patient with Cushing’s disease died in the further course from late rupture (1 week after surgery) of a postoperative pseudoaneurysm at the internal carotid artery. Of the 239 adenomas, comprising Knosp grades 0–4, 178 (74%) were considered completely resected and the resection of 61 (26%) was intended to be incomplete according to the surgeon’s impression at the end of the surgery. The strongly differing results for each Knosp grade are shown in Table 2. A follow-up that could be gathered for 154 patients (64%) after a median follow-up time of 6.75 months mostly confirmed those results. For the evaluation of a remission in functioning adenomas, the chemical parameters were used, whereas a remission for the non-functioning adenomas was defined by lack of tumor remnant in the MRI. Comprising all Knosp grades, a remission was found in 69.4% of the patients (Table 3).

**Table 2:** Resection rate of adenomas treated with the exoscope (estimated by the surgeon)

		Microadenomas, *n* = 78			Macroadenomas, *n*= 161		
Knosp		GTR	PR	Surgical pretreatment	GTR	PR	Surgical pretreatment
0	*n*=54	46	-	9	8	-	1
1	*n*=69	28	-	6	36	5	3
2	*n*=49	1	1	-	34	13	5
3	*n*=39	1	1	2	22	15	7
4	*n*=28	-	-	-	1	27	11

GTR gross total resection, PR partial resection

**Table 3:** Resection rate of adenomas treated with the exoscope at time point of follow-up (median 6.75 months)

		FPA				NFPA			
		Micro, *n* = 42		Macro, *n*= 49		Micro, *n* = 0		Macro, *n*= 63	
Knosp		GTR	PR	GTR	PR	GTR	PR	GTR	PR
**0**	*n*=29	19	4	4	1	-	-	1	-
**1**	*n*=46	12	4	15	2	-	-	13	-
**2**	*n*=34	1	1	5	5	-	-	18	4
**3**	*n*=24	-	1	6	1	-	-	11	5
**4**	*n*=21	-	-	-	10	-	-	2	9

GTR gross total resection, PR partial resection, FPA functioning pituitary adenoma, NFPA non-functioning pituitary adenoma, Micro microadenoma, Macro macroadenoma

Intraoperatively, 49 CSF leakages occurred (mostly due to subarachnoidal location) and were repaired with autologous muscle transplantation from the right lateral vastus muscle in the same sitting. In the further course, two of these patients developed postoperative CSF leakages requiring three surgical revisions overall. No technical failures, no restrictions using the instruments, and no limitations in the range of view occurred intraoperatively or forced to end surgery. In the postoperative evaluation, the individual surgeon’s impression was positive with one restriction due to a possible diagonal camera view which could lead to missing the lateral orientation without navigation or anatomical landmarks for the unexperienced user. The consensus of advantages and disadvantages are shown in Table 4.

**Table 4:** Comparison of advantages/disadvantages of the exoscope compared to the microscope

Advantages	Disadvantages
Small instrument	The flexibility of the camera’s viewpoint bears the risk of deviation from the midline without landmarks or navigation
Relaxed sitting position	
High-resolution enlargement/zoom and therefore better lateral vision through the mirrors	
Flexible and fast attitude and adaption	
Fast learning curve	
Good for overweight patients	
Fast visualization of vessels	
Outstanding for teaching	

### Learning curve

Looking at the learning curve in the exoscope group, we extracted the non-invasive microadenomas (Knosp 0+1) without prior surgery (*n*=61) and compared the corresponding nine cases out of the first 50 surgeries with the 52 out of the remaining 246 surgeries. All had hormonally active adenomas and were all evaluated concerning their postoperative biochemical remission. We discovered that the mean duration of surgery could be reduced with increasing experience and expertise in usage of the exoscope (mean 1:23 h vs. mean 1:10 h). There was an increase of remission rate after finishing the learning curve (Table 5).

**Table 5:** Comparison of the groups exemplary by non-invasive microadenomas (Knosp 0+1) without prior surgery

	Exoscope		Microscope
	9 patients‚ learning curve—first 50 surgeries	52 patients, out of the remaining 246 surgeries	52 patients, out of all surgically treated patients between Jul 2017 and Aug 2018
Median age (years)	41	44	41
Sex w/m	2:1 (6/3)	2.5:1 (37/15)	4.2:1 (42/10)
Median duration	1:23 h	1:10 h	1:10 h
Adenoma type (*n*)	PRL 1, ACTH 4, STH 3, TSH 1	PRL 10, ACTH 27, STH 14, TSH 1	PRL 13, ACTH 25, STH 14
Failed initial postop. chemical remission	1 × Cushing’s disease, 1 × acromegaly, 1 × TSHoma	3 × Cushing’s disease, 1 × acromegaly	3 × Cushing’s disease, 1 × prolactinoma (with neuroleptic medication)
Complications	-	-	2 CSF leakages (3.8%)

### Exoscope vs. microscope

Comparing exoscopic and microscopic surgeries, we extracted 52 consecutive patients with non-invasive microadenomas without prior surgery from our microscopically treated patient collective (between July 2017 and August 2018). There was no difference regarding duration of surgery between the exoscopic and microscopic cohorts and, again, there was no significant difference regarding remission rate. In the microscopically treated patient cohort, two CSF leakages occurred (3.8%), compared to no CSF leakage in the exoscopic group (Table 5).

## Discussion

The feasibility of a new video microscope, 4K 3D exoscope (Orbeye, Olympus), was proven for endoscope-free transsphenoidal pituitary surgery. The team quickly adapted to the new setup, working position, and viewing angle. The already reported [1] enhanced surgeon’s comfort due to the ocular-free, head-up working position was immediately accepted and reached high consensus in the evaluation. To have all axes in line results in an exceptionally comfortable position for the surgeon and reduces the risk of fatigue and bad posture. The forward-facing position helps with the coordination and makes for a more intuitive way of work. Our surgeons did not experience any discomfort like nausea or fatigue while using the 3D glasses. This might be due to a more static visual field during surgery, as opposed to the 3D experience, e.g., in cinema, where pictures are moving rapidly. Crossfade and darkening of the visual field may occur in case of bright tissue or bleeding, respectively; this can easily be compensated by adapting the light intensity using the foot pedal.

The two surgeons have both had sufficient experience in microscopic pituitary surgery before switching to the exoscope. The subjective learning curve of those two surgeons was exceptionally steep. Another surgeon currently in training with the exoscope is experiencing a similarly steep learning curve without relevant microscopic operating experience in pituitary surgery. Nevertheless, a short learning curve of 20–30 surgeries for each surgeon is recommended to become completely familiar with this new workflow. This might be the reason why earlier attempts to utilize this new technology were not successful. Earlier reports referred to small case series with exoscope-using sequences of less than 5 min in a procedure lasting 85 min [14].

Here, we show that a great variety of intra- and suprasellar pathologies, including the most common ones, can successfully be operated on with the 4K 3D exoscope transsphenoidally. The high optic quality offers a safe and high resection grade for intrasellar tumors. Even extensive lesions could be managed without major difficulties in a monoportal approach diminishing soft tissue preparation of the nose to a minimum. As a result, we consider the 4K 3D exoscope as safe as with the microscope, which has been objectified by a complication rate that has not increased compared to the equivalent microscopic cohort. Additionally, the similar duration of surgery for adenomas compared to the selected microscopic cohort as well as similar resection rates shows at least equivalent feasibility.

**Fig. 2:**
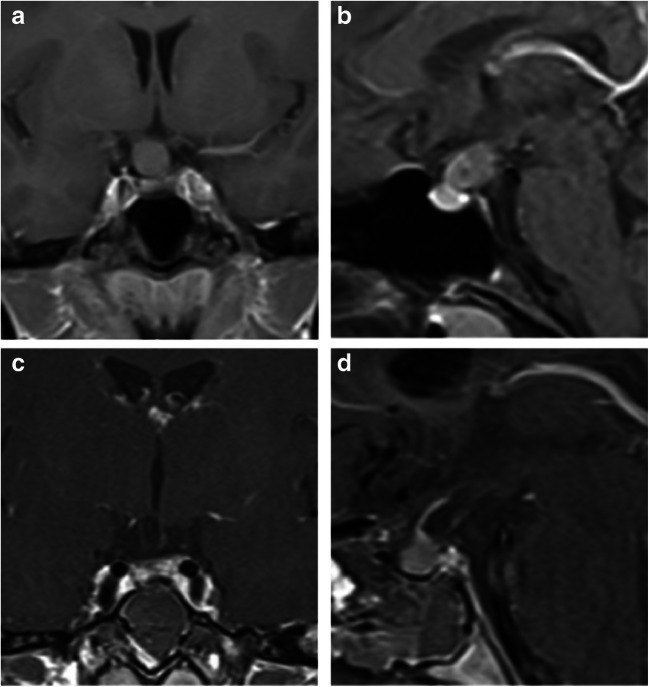
Suprasellar appoach in a Rathke’s cleft cyst in a young female with visual deterioration as a case study. Shown are the preoperative MRI in the coronal plane (**a**) and sagittal plane (**b**) and the postoperative MRI in the coronal plane (**c**) and sagittal plane (**d**). The cyst content was able to be completely removed with a large fenestration of the cyst’s membrane and no observation of hormonal dysfunction upon further clinical course

**Fig. 3:**
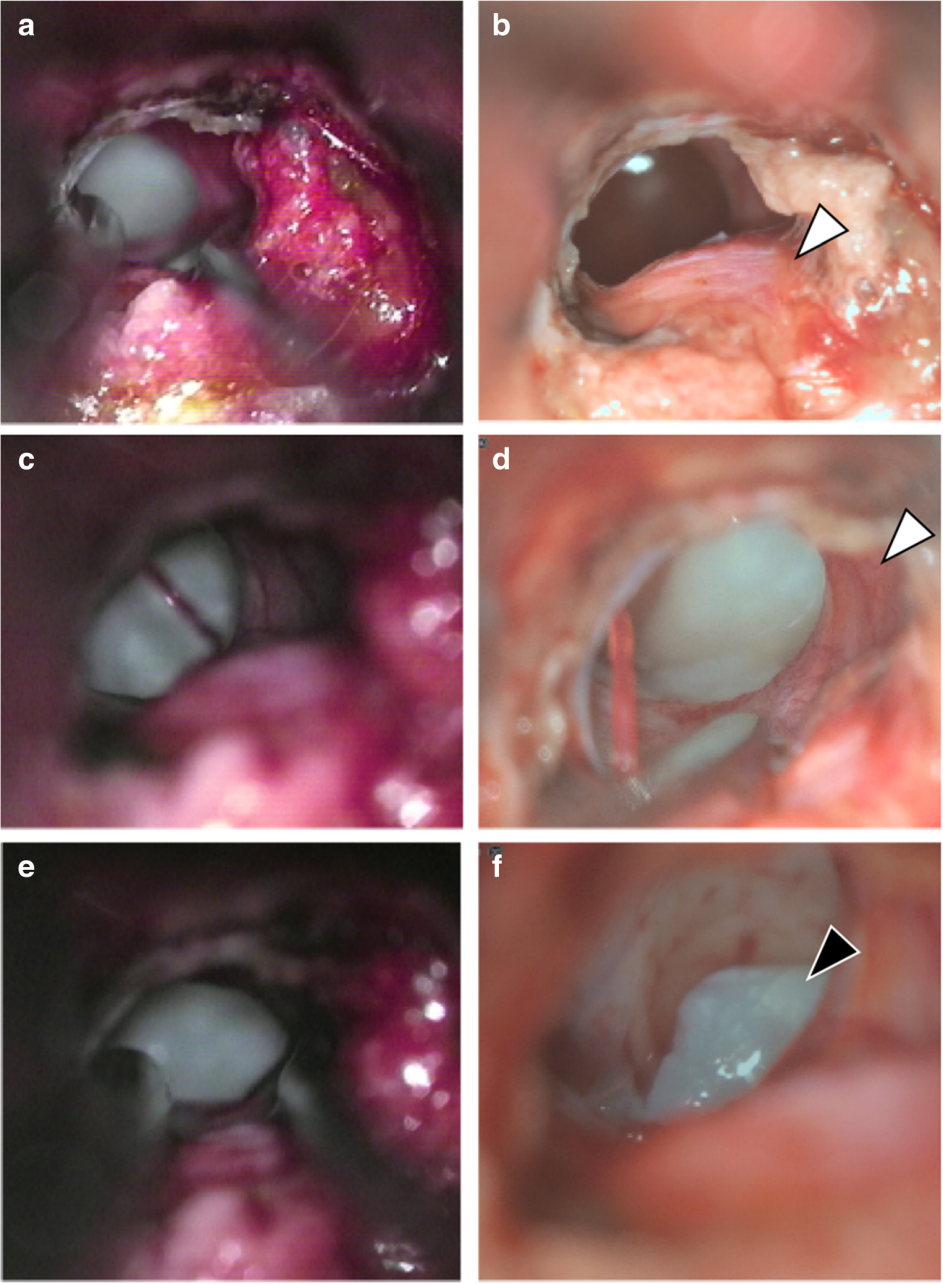
Comparison of the surgical microscope (Zeiss S7) and the Orbeye 4K 3D video microscope. Representative images of the presented case study with a suprasellar approach in a Rathke’s cleft cyst in a young female with visual deterioration with the focus on the dura/anterior pituitary and pituitary stalk (white arrowheads) and the cyst content (black arrowhead). The images taken with the surgical microscope are shown left (**a**, **c**, **e**) and the images taken with the Orbeye are shown on the right side (**b**, **d**, **f**) in 2D

In certain aspects, the 4K 3D exoscope appears superior to the microscope: it provides a better image, as also reported for other neurosurgical procedures [13]; the small footprint is beneficial especially in smaller operating rooms; the excellent resolution and the combination of optical and digital zooming enable an enlargement of the surgical field that is beyond the microscope’s magnification capacities. An illustrative case of transsphenoidal surgery (microscopic versus exoscopic view) can be seen in Figs. 2 and 3. Here, you can get an impression of the different optical view between a microscope (Zeiss S7) and an exoscope (Orbeye).

For demonstration and teaching purposes, as well as for transmission live surgery, it is highly recommendable as the spectator experiences the same view as the surgeon.

The only disadvantage that reached consent was the risk of deviation from the midline without landmarks or navigation due to the flexibility of the camera’s viewpoint. The possibility of a diagonal camera view might be considered a disadvantage for surgeons with little experience in exoscopic surgery; however, after a certain learning curve, it can turn into an advantage due to a more flexible and wider view of the surgical field. By orientating on the anatomical landmarks (septal vomer/sphenoid septum/carotid canal), this slight difference in use in comparison with the microscope can be fully compensated. In difficult cases, it might be helpful to add neuronavigation.

Currently, the exoscope (Orbeye) does not support a navigated focal point and integrated augmented navigation in the surgical view. Nevertheless, in other neurosurgical specializations, the neuronavigation is reported to be feasible with this exoscope providing a satisfying workflow using the pointer (oral communication). The image of the navigation system can either be projected picture-in-picture on the surgeons’ monitor or build a panel of multiple monitors. In contrast to the microscope, there are three major differences: (1) the operator does not have to look away from the ocular lens of the surgical microscope to look onto the monitor of the navigation system. It is the opposite: with the exoscope the surgeon is looking on the monitors all the time and therefore does not need to refocus and adjust the eyes. (2) The small camera head does not block the signal for the navigation as it often happens with the surgical microscope. (3) There is a lot of space in the surgical distance for precise navigation with a pointer or a navigated instrument (e.g., navigated suction). This function could not entirely be assessed during this study since navigation is not included in our standard procedure workflow of transsphenoidal pituitary surgery. The experience outlined here is based on experiences in the cranial neurosurgery team of the same hospital. Neuronavigation is performed differently across the neurosurgical community which is why this functionality should be individually assessed with the preferred setup.

The initial costs for purchasing the Orbeye exoscope is equivalent to a state-of-the-art neurosurgical microscope with similar configuration. The modularity of the exoscopic system allows an adjustment to the individual requirements and budget and adding features at a later point. The hybrid solution of a standard surgical microscope with an exoscope module exceeds the price of this purely digital exoscope. The operating costs are comparably low since the only consumables are the sterile drapes. No other consumables are required and no parts have to be exchanged in regular turns. Different service plans are available for the Orbeye. The costs are competitive to the standard surgical microscope since the LED light source is maintenance-free (more than 10,000 hours life span) and a design which is focused on simplicity and a reduced amount of moving parts.

In our test, the system has been proven robust and durable. No major damage is reported within the use of more than 2 years. The system performed reliably without system failure. In one case, the image guiding cables got damaged because of improper handling. This caused a loose contact and disruption of the image. This seems to be caused rather by inexperienced personnel than by improper design and manufacturing because it did not appear at a later stage of the learning curve. We consider the risk of a malfunction to be at the same level as with the standard surgical microscope.

The system provides several emergency measures, most importantly a backup light source. In case of a complete failure, the Orbeye comes with an optical loupe which can be attached to the camera head in order to proceed with the microsurgery.

In comparison with existing techniques, the Orbeye definitely contributes by filling a gap between the microscope and endoscope. Its use shows similarities to that of the endoscope, such as better resolution and a better viewing angle by looking onto a screen. And it remains a single surgeon procedure even for extended tumor growth. Additionally, due to the larger distance of the camera to the surgical site, there is no contamination and image deterioration due to blood on the lens during surgery.

Since in our collectives there were less CSF leakages in the exoscopic cohort than in the microscopic cohort, it might also indicate a possible advantage of endoscopy due to a higher CSF leakage rate for endoscopic procedures compared to microscopic surgery in literature [3].

On the other hand, even though the exoscope might be superior to the microscope regarding lateral visualization in extensive tumor growth, there are certainly limitations compared to the endoscope in terms of lacking dynamic motion and angled lenses. To overcome the disadvantage of less lateral visualization, we use mirrors and a suction-irrigation-device with straight and angled tips. Also, the addition of the endoscope can be considered. Split screen mode offers the complementary benefit of the endoscope instead of mirrors which may increase the possibilities of lateral view but has to be evaluated in comparison to endoscopic transsphenoidal procedures in the next step.

The real difference in outcome should be delineated in future comparative studies with a focus on the comparison of endoscope vs. Orbeye including endoscopic inspection rather than endoscope vs. microscope.

## Conclusion

The Orbeye exoscope presents with optical and digital zoom options as well as a 4K image resolution and 3D visualization resulting in better depth perception and flexibility in comparison to the microscope. After a short learning curve, this 4K 3D exoscope quickly replaced the standard microscope in our operating room. After the advent of exoscopes [6], we think this 4K 3D exoscope represents the next developmental step in microscopic transsphenoidal pituitary surgery. This new technology is well suitable for this approach and combines the benefits of a classical microscope with some amenities of an endoscope.

## Abbreviations

4K: description of media resolution
2D/3D: two dimensional/ three dimensional
LED: light-emitting diode
J. F.: Jörg Flitsch
R. R.: Roman Rotermund
CSF: cerebrospinal fluid
GTR: gross total resection
PR: partial resection
